# Investigation of Fast-Charging and Degradation Processes in 3D Silicon–Graphite Anodes

**DOI:** 10.3390/nano12010140

**Published:** 2021-12-31

**Authors:** Yijing Zheng, Danni Yin, Hans Jürgen Seifert, Wilhelm Pfleging

**Affiliations:** Institute for Applied Materials-Applied Materials Physics (IAM-AWP), Karlsruhe Institute of Technology (KIT), Hermann-von-Helmholtz-Platz 1, 76344 Eggenstein-Leopoldshafen, Germany; danni.yin@hotmail.com (D.Y.); hans.seifert@kit.edu (H.J.S.)

**Keywords:** fast-charging, silicon anode, graphite anode, laser structuring, LIBS, 3D battery

## Abstract

The 3D battery concept applied on silicon–graphite electrodes (Si/C) has revealed a significant improvement of battery performances, including high-rate capability, cycle stability, and cell lifetime. 3D architectures provide free spaces for volume expansion as well as additional lithium diffusion pathways into the electrodes. Therefore, the cell degradation induced by the volume change of silicon as active material can be significantly reduced, and the high-rate capability can be achieved. In order to better understand the impact of 3D electrode architectures on rate capability and degradation process of the thick film silicon–graphite electrodes, we applied laser-induced breakdown spectroscopy (LIBS). A calibration curve was established that enables the quantitative determination of the elemental concentrations in the electrodes. The structured silicon–graphite electrode, which was lithiated by 1C, revealed a homogeneous lithium distribution within the entire electrode. In contrast, a lithium concentration gradient was observed on the unstructured electrode. The lithium concentration was reduced gradually from the top to the button of the electrode, which indicated an inhibited diffusion kinetic at high C-rates. In addition, the LIBS applied on a model electrode with micropillars revealed that the lithium-ions principally diffused along the contour of laser-generated structures into the electrodes at elevated C-rates. The rate capability and electrochemical degradation observed in lithium-ion cells can be correlated to lithium concentration profiles in the electrodes measured by LIBS.

## 1. Introduction

The lithium-ion battery (LIB) was commercialized by Sony in 1991 [1]. Nowadays, the LIB has become a common and important energy storage device for mobile devices. In addition, due to its high gravimetric and volumetric energy density, the LIB has been a standard energy source for electric vehicles and drones [2]. The production cost of lithium-ion batteries (LIBs) per kilowatt-hour was USD 764 in 2009–2010 and could already be reduced to USD 111 in 2020 [3,4]. A further reduced cost of USD 92 was forecasted in 2025 [5]. The further development and establishment of hybrid and electric vehicles (xEV) are closely linked to the next generation of LIBs, which are characterized by further increased power density as well as high energy density [6]. In the current research, the main goal is to conceive cells with an energy density of about 350–500 Wh/kg at the cell level [7]. In addition, a short charging time (<15 min) and a range of over 600 km with a single battery charge have been aimed so that the electric drive system with LIBs can offer a comparable range and “fuelling time” as the combustion engine. For this purpose, there are considerable requirements for development at the cell and the material level. Thus, further development of new materials for anode and cathode with high specific capacity is necessary.

In terms of material development, silicon has been considered as a next-generation anode material due to its theoretical capacity of 3579 mAh/g, which is significantly higher than that of the commonly used graphite (372 mAh/g) [8]. Silicon provides a moderate potential of 0.2–0.3 V (vs. Li/Li^+^), and lithiated silicon is more stable in liquid electrolytes than lithiated graphite [9]. However, the high specific capacity leads to an enormous volume change of silicon up to 300% during lithiation and delithiation, which is a well-known challenge for applications and commercialization of silicon-based anodes. The volume change causes crack formation in the silicon particles, and subsequently the solid electrolyte interphase (SEI) layer is continuously rebuilt. The internal impedance of the cells increases with increasing SEI layer thickness [10,11]. The repeated particle cracking leads to pulverization, electrical insulation of the silicon, and film delamination [12].

Therefore, the main challenges for the commercialization of silicon-based anode materials are to improve their chemical and mechanical integrity in order to achieve application-related lifetime, cycling stability, and capacity retention [13]. In the last decades, several strategies have been developed with respect to the reduction of compressive stress in silicon-based electrodes. Silicon 3D nanostructures, including nanowires [14,15], porous/hollow structures [16,17], and silicon-based composite films [18,19], as well as silicon composites with 3D structural designs [20,21], have shown improved cycling stability and rate capability compared to 2D electrode designs. However, recent research results have been successful but are still far from the practical requirements of consumer and commercial mass production. Therefore, a combined utilization of graphite and Si powders has been introduced in this work. Graphite has a low specific capacity but can provide improved volumetric energy density and cycle stability as well as a long cycle lifetime (>80% of initial capacity for more than 500 cycles). The silicon contributes to the increased specific capacity of the entire composite electrode. Graphite is a common anode material that has already been successfully commercialized. For a combination of these types of active materials, conventional battery production can be adapted quickly and cost-effectively [22,23]. 3D electrode architectures achieved by ultrafast laser structuring was successfully implemented in batteries on a laboratory scale [24,25]. Previous works [25] revealed that mechanical stress within the silicon–graphite (Si/C) electrodes can be significantly reduced by applying 3D architectures, and these cells can achieve an improvement concerning rate capability, cycle stability, and cell lifetime.

This work is focused on the investigation of lithium concentration profiles related to fast charging and discharging, as well as cell degradation mechanism in structured and unstructured Si/C electrodes. For this purpose, laser-induced breakdown spectroscopy (LIBS) was employed, and electrochemical properties of silicon and Si/C electrodes were analyzed by cyclic voltammetry (CV) and galvanostatic measurements (GM).

## 2. Materials and Methods

### 2.1. Preparation of Electrode Material

The silicon–graphite electrodes consisting of 10–20 wt% silicon were fabricated for galvanostatic measurements and subsequent laser-induced breakdown spectroscopy (LIBS). The mass of active materials, silicon, and graphite is kept to 80 wt% of the total electrode mass. The various chemical composition of electrode materials, thickness, and the corresponding application are listed in Table 1. To avoid the crack formation of silicon particles during electrochemical cycling, we applied the nano-sized silicon particles with an average particle size of 150 nm (2W iTech, LLC, San Diego, CA, USA). After exposure to the ambient air, a native oxide layer was built on the silicon surface. The chemical properties of the silicon powder were analyzed by inductively coupled plasma-optical emission spectrometry in advance. The used silicon powder contains 94 wt% silicon and 3.84 wt% oxygen. Silicon particles were mixed with flake graphite particles ( $D_{50}$= 15.26 µm, Targray, Kirkland, QC, Canada) and carbon black (Timcal Super C65, MTI Corporation, New York, NY, USA) in pre-prepared sodium carboxymethyl cellulose (Na-CMC, MTI Corporation, Richmond, CA, USA) solution. A planetary ball milling machine (Pulverisette 7 premium line, Fritsch GmbH, Idar-Oberstein, Germany) enables the achievement of a homogenous slurry without micro-sized silicon agglomerates. Styrene-butadiene rubber (SBR, MTI Corporation, Richmond, CA, USA) can contribute to the improvement of the mechanical stability of electrode and film adhesion. Hence, 5 wt% styrene-butadiene rubber was added at the end of the mixing process.

The prepared slurry was coated on a nickel-coated copper current collector (Targray, Kirkland, QC, Canada) by tape casting. The nickel layer has a thickness of 1-2 µm and serves to improve the film adhesion. Afterwards, the electrode was dried at room temperature for 2.5 h. Prior to laser structuring, the electrode was calendered using a hot rolling press machine (HR01 Hot Rolling Machine, MTI Corporation, Richmond, CA, USA). The porosity of the electrode is related to its thickness and can be calculated by the ratio of hollow volume to the total volume of the electrode using material density. The used equation for the calculation is described elsewhere [26]. The electrode was passed several times through two counter-rotating rollers with gradually reduced roller gap distance. The prepared electrodes have a porosity of about 42%.

### 2.2. Ultrafast Laser Processing

A processing system (PS450-TO, Optec, Frameries, Belgium) with a pulsed femtosecond laser (Tangerine, Amplitude Systèmes, Bordeaux, France) was used to create 3D structures on the surfaces of the anodes and cathodes and to cut out the electrodes. The femtosecond laser operates at a fundamental wavelength of 1030 nm with a pulse duration of 350 fs. The pulse repetition rate of 200 kHz and 500 kHz were applied for cutting and structuring, respectively. With respect to isotropic volume expansions during lithiation in the silicon–graphite electrode, grid structures with a pitch distance of 100 µm and 300 µm were generated on the electrodes.

### 2.3. Cell Assembly

Coin cells of type CR2032 (MTI Corporation, Richmond, CA, USA) were used to characterize the electrochemical properties of the laser-structured (“LS”) and unstructured (“reference”) electrodes. Coin cells were also used for calibration samples regarding laser-induced breakdown spectroscopy. For providing a clear type of abbreviation, the cells are denoted as “LS cells” and “reference cells”. Cell assembly was performed in a glove box filled with argon (LABmaster SP, MBraun Inertgas-Systeme GmbH, Munich, Germany) in a high-purity atmosphere (O_2_ < 0.1 ppm, H_2_O < 0.1 ppm). The electrodes examined have a diameter of 12 mm. Lithium foil (Sigma Aldrich, St. Louis, MO, USA) with a thickness of 250 µm and a diameter of 11 mm was used as a counter electrode. A glass fiber separator with a diameter of 15 mm (GF/A filter, thickness 260 μm, Whatman, Maidstone, UK) served as an electrical insulator between the electrodes. The electrolyte used (LP57, Merck AG, Darmstadt, Germany) consists of a 1.3 mol/L lithium hexafluorophosphate (LiPF_6_) in ethylene carbonate (EC) and ethyl methyl carbonate (EMC) solution with a weight ratio of 3:7. Additionally, 5 wt% fluoroethylene carbonate (FEC) was added to the mixture, which can contribute to the formation of a more stable solid electrolyte interphase (SEI). A total of 120 µL electrolyte was added to the electrode and separator. To ensure a homogeneous wettability of the electrodes with the liquid electrolyte, the thick electrodes (thickness L > 100 µm) were previously immersed in the electrolyte bath for 30 min. The cell components were assembled using an electric coin cell crimper (MSK-160D, MTI Corporation, Richmond, CA, USA). 

### 2.4. Electrochemical Analyses

The galvanostatic measurements were performed at room temperature 22 °C by a battery tester (BT 2000, Arbin Instruments, College Station, TX, USA). After cell assembling, the formation was carried out. The half-cells with Si/C electrodes were discharged at a low constant current (cc) of 0.02C up to cutoff voltage (0.01 V). A constant voltage (cv) was applied just after the cell reached the cutoff voltage. The cv step was completed when the measured current at the cell was less than a specified cutoff current. Subsequently, the cells were charged at the same C-rate to upper cutoff voltage (1.5 V). The procedure was repeated for three cycles to ensure a homogenous SEI formation. After formation, the cells were prepared for the subsequent electrochemical analyses.

For rate tests, the cells were charged and discharged with increasing C-rate up to 2C and then with the lower C rate of 0.1C to investigate rate capability and cell degradation.

For a quantitative evaluation of the lithium concentration at any Si/C electrode, a calibration is necessary. Identical structured Si/C electrodes with a pitch distance of 300 µm were prepared for this purpose. After formation, the cells were discharged to the selected voltages (Table 2) using 0.1C. Subsequently the voltage was kept constant for further 3 h.

Cyclic voltammetry, which is applied to investigate the phase change in redox reactions, is carried out directly after cell assembling by a potentiostat VMP-3 (BioLogic Science Instruments, Seyssinet-Pariset, France). Formation of crystalline phase Li_15_Si_4_ (cr-Li_15_Si_4_) was studied with different voltage ranges of 0.01–1.5 V and 0.05–1.5 V. A sweep rate of 10 µV/s was used for recording the current response. 

### 2.5. Laser-Induced Breakdown Spectroscopy and Creation of Calibration Data

Laser-induced plasma spectroscopy (LIBS, type: FiberLIBS SN013, SECOPTA analytics GmbH, Teltow, Germany) enables the analysis of the lithium distribution in the electrodes. A passive-mode-locked Nd:YAG laser with a wavelength of 1064 nm, pulse duration of 1.5 ns, and repetition rate of 1–100 Hz was integrated in the LIBS system. The laser beam was focused on the sample surface with a diameter of about 100 µm, which also corresponded to the lateral resolution of LIBS measurements. The measurements were performed with a maximum pulse energy of 3 mJ and a laser pulse frequency of 100 Hz. The analyzed material was ablated by laser pulses. Meanwhile, laser-induced material vapor plasmas were generated. As the plasmas decayed, the element-specific spectra were emitted. The emitted spectra were recorded by a Czerny-Turner spectrometer (FiberLIBS SN013, SECOPTA analytics GmbH, Teltow, Germany) in combination with two Hamamatsu S11155 charge-coupled detectors (CCD) (Hamamatsu Photonics K.K., Hamamatsu, Japan). By means of this setting, the emission spectra in the range of 229–498 nm and 569–792 nm can be analyzed by one single laser pulse.

The software SecViewer (version 1.9, SECOPTA analytics GmbH, Teltow, Germany) was used to evaluate the spectra. The characteristic wavelengths of the elements relevant for the Si/C electrodes can be assigned. The intensity of the characteristic wavelength allows for a qualitative analysis of the element distribution in an electrode. Due to the effect of self-absorption at the wavelength of 670.77 nm, the Li^I^ emission wavelength of 610.35 nm was used for the analysis of lithium [26].

For a quantitative evaluation of lithium concentration in the type of Si/C electrodes, a calibration file related to the used LIBS device is necessary. Structured Si/C electrodes with 10 wt% silicon in different lithiated states, as listed in Table 2, were prepared for this purpose. Hereby, grid structures with a pitch of 300 µm were applied to improve the reproducibility of the electrochemical titration procedure. Subsequently to titration, these prepared cells were disassembled in the glove box. The disassembled electrodes were immersed in a dimethyl carbonate (DMC) solution for 30 min and then washed up with a fresh DMC solution. This cleaning step was repeated twice to avoid contamination from the residual lithium salt by the used electrolyte. Thereafter, half of the electrode was used for the establishment of the calibration by LIBS and the other part of the electrode was used for the determination of the element concentration, e.g., lithium, silicon, and carbon, by applying inductively coupled plasma-optical emission spectrometry (ICP-OES, OPTIMA 4300 DV, PerkinElmer, Waltham, MA, USA). The calibration data were derived from LIBS and ICP-OES as described in [26]. Lithium concentration and distribution related to C-rates was systematically investigated in the structured and unstructured Si/C electrodes. The electrochemical cycling procedure for each type of electrodes is shown in Table 3.

## 3. Results

### 3.1. Laser Structuring

**Figure 1:**
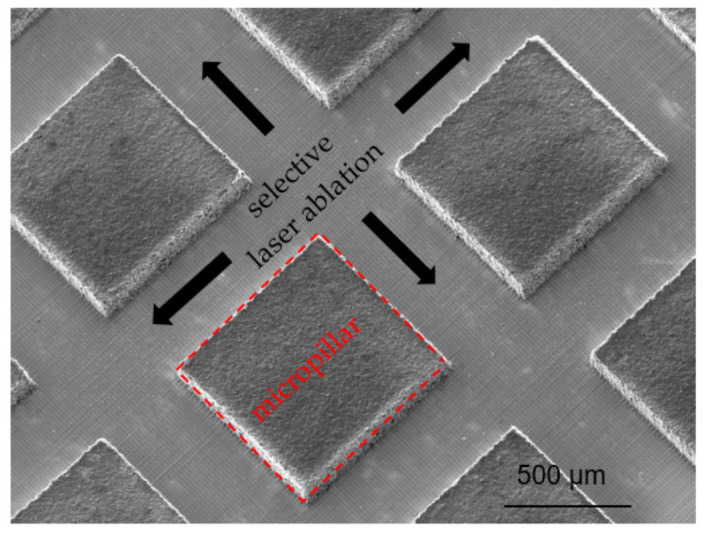
Scanning electron microscopy (SEM) image of a laser-generated model electrode with micropillar structures (800 µm × 800 µm).

Model electrodes with micropillars were designed for the LIBS measurements to investigate the lithium diffusion pathway in the structured electrodes. These special electrodes consist of micropillars with dimensions of 800 × 800 µm^2^ (Figure 1), which are arranged at a distance of 1200 µm from each other. The microcolumns are realized by direct laser ablation of the electrode material around the micropillars. The layer thickness of the electrodes is about 100 µm. A smooth copper surface reveals a successfully selective ablation without damaging the copper current collector.

**Figure 2:**
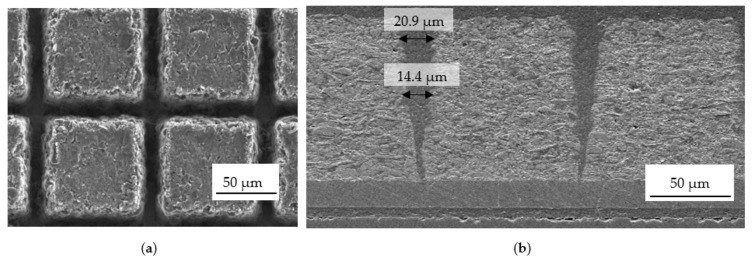
SEM images of laser-generated grid structures with a period of 100 µm: (**a**) top view and (**b**) cross section.

When the necessity of a small material loss after laser structuring is taken into account, narrow channels on the electrodes are typically required. The structured Si/C electrode is demonstrated in Figure 2 in the top view (a) and cross-section view (b). The structured channels in thick electrodes (>70 µm) usually revealed a “V”-shaped contour. The channel widths on the top and in the middle were 21 ± 2 µm and 14.4 ± 1 µm, respectively, and its value reduced to a few micrometers on the bottom of the channel close to the current collector.

### 3.2. Electrochemical Properties of Silicon-Based Electrodes

#### 3.2.1. Cyclic Voltammetry of Cells with Silicon Electrodes

**Figure 3:**
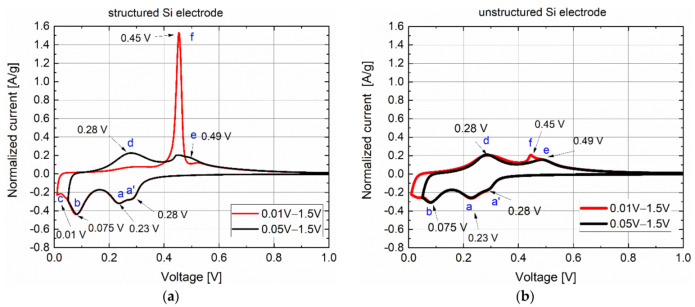
Cyclic voltammogram of cells with structured (**a**) and unstructured (**b**) Si electrodes recorded by a swap rate of 10 µV/s in a voltage window of 0.01–1.5 V and 0.05–1.5 V.

The phase change of graphite has already been studied in detail [27,28] by employing (CV). Concerning the silicon–graphite composite system, it is necessary to study the material property of silicon separately. Therefore, cyclic voltammetry was performed on the cells with thin composite silicon anodes consisting of 40 wt% silicon (type 1 in Table 1) directly after the cell assembly. It is well known that the metastable crystalline phase Li_15_Si_4_ is only formed when the cutoff voltage is lower than a certain voltage value. Therefore, two cutoff voltages of 0.01 V and 0.05 V were set up for CV. The cyclic voltammograms of cells with the LS and reference electrodes recorded from the third cycle are shown in Figure 3 using a normalized current I_Norm_ (I_Norm_ = measured current/mass of active materials) as a function of the voltage. The alloying process (lithiation) of silicon occurs for voltages lower than 0.3 V. For all cells, two broad current maxima (a and b) appeared at 0.23 V and 0.075 V, respectively. A secondary maximum a’ was detected at 0.28 V, as in [29], marking the formation of the P-I phase (LiSi). Finally, the phase transition from P-I to P-II (a-Li_7_Si_3_) occurred at 0.23 V. The second broad current maximum at 0.075 V can be attributed to the formation of the P-III phase (a-Li_3,16_Si). Whether a-Li_3.5_Si [30] or Li_3.16_Si [29] is formed in this voltage range is still debated in the literature. Up to 0.05 V, no different current profile was observed for the LS and reference cells during lithiation. A current maximum c at 0.01 V could be detected at the LS cells, indicating the formation of cr-Li_15_Si_4_. However, the voltage values of the current maxima c and a slightly differed from the data from [29,31,32].

During the subsequent delithiation process in a voltage window of 0.01–1.5 V, a current maximum f with high current density (1.5 A/g) appeared at 0.45 V from LS cells. This current maximum and the corresponding voltage were in good agreement with data from the literature [13,31,33] and describe the delithiation of the crystalline phase cr-Li_15_Si_4_. Two broad current maxima d and e were observed at 0.28 V and 0.49 V, respectively, when the lower cutoff voltage was increased to 0.05 V. In this case, the current maxima f at 0.45 V was significantly reduced but still discernible and overlapped with the broad peak e at 0.49 V. This indicates that the amorphous phase Li_x_Si was not completely converted to cr-Li_15_Si_4_ during the cathodic reaction at the lower cutoff voltage of 0.05 V. Probably due to the weak current maximum, only a small amount of cr-Li_15_Si_4_ was formed at 0.05 V. The current maximum at 0.28 V indicated the phase transformation from a-Li_3.5_Si to a-Li_7_Si_3_, and the peak at 0.49 V indicated the transition from Li_7_Si_3_ to LiSi.

Figure 3b demonstrates the cyclic voltammogram of the reference cells with unstructured Si electrodes. In comparison to LS cells, the reference cells exhibited a similar cyclic voltammogram as a function of voltage during the cathodic reaction (lithiation). No current maximum was detected at the voltage of 0.01 V. The clear difference in current peaks was observed in the anodic reaction (delithiation). A weakly pronounced current peak f of 0.2 A/g can be seen in comparison to the measurement with LS cells (1.5 A/g). This current maximum f was completely extinguished in the smaller voltage range (0.05–1.5 V). The current peak at 0.45 V recorded from reference was significantly smaller than those of the cells with LS electrodes. It was determined that the crystalline phase could not be formed effectively in the reference cells, even if the lower cutoff voltage was set to values smaller than 0.05 V.

#### 3.2.2. Galvanostatic Measurements of Cells with Si/C-Electrodes

**Figure 4:**
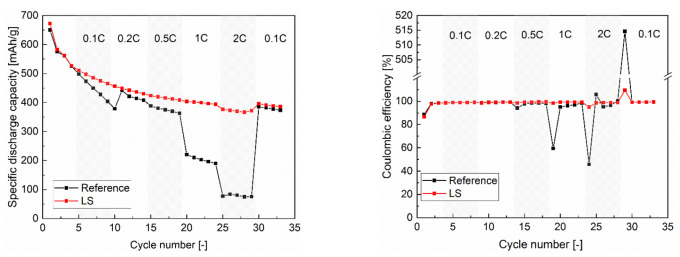

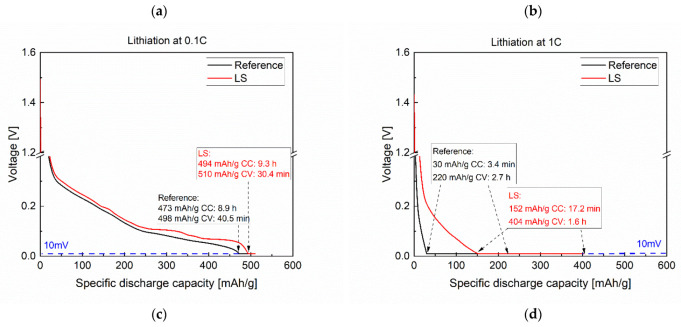
Results from galvanostatic measurements of cells with structured (LS) and unstructured (reference) electrodes: (**a**) specific discharge capacity as function of C-rate in rate capability tests, (**b**) corresponding coulombic efficiency, (**c**) voltage drop as function of specific discharge capacity by applying 0.1C, and (**d**) 1C (cccv-testing procedure was applied during the discharging process).

Rate capability and cell degradation were studied in coin cell design using high-energy silicon–graphite electrodes (type 4 in Table 1). Grid structures with a pitch distance of 100 µm (shown in Figure 2) were applied to improve rate capability. Figure 4a,b show the selected results of the discharge capacity (lithiation of the Si/C electrode) and the corresponding coulombic efficiency as a function of cycle number. The coulomb efficiency (CE) obtained from the first cycle, which describes the ratio of removed charges to previously stored charges during a charge–discharge process in a cell, was about 84% for reference and LS cells. After formation, all cells were symmetrically cycled with step-wise increased C-rate. The discharge capacity of LS cell slightly dropped with increased C-rate and the cycle number and could provide a capacity over 380 mAh/g at 2C. Subsequently, the capacity increased to over 400 mAh/g at 0.1C, which is lower than the initial capacity of 480 mAh/g. This capacity fading can be attributed to the thickness of the electrode, the chemical degradation, side reaction with electrolyte, and quality of the used silicon depending on the supplier. In comparison to the previous research in [25], the silicon-containing high oxygen content (14 wt%) revealed a reduced initial capacity loss during electrochemical cycling. The natural SiO_2_ layer on the surface of silicon particles might prevent the cracking of silicon particles and subsequent rebuilding of SEI layers. However, the coulombic efficiency of LS cell maintained about 100%. A deviation from 100% was observed at the first cycle by charging C-rate to 2C and to 0.1C, which indicated cell polarisation.

By comparison, the reference cell revealed an unstable discharge capacity and coulombic efficiency. For C-rates equal and above 1C, a significant capacity fading (from about 200 mAg/h at 1C down to less than 100 mAg/h at 2C) can be observed. The coulombic efficiency was reduced to less than 50% at the first cycle by charging C-rate. In this case, the inserted lithium in the Si/C electrode could not be completely removed back to the counter electrode due to the applied high C-rate and resulting polarisation. Furthermore, an extremely high value of over 515% was obtained when the reference cells cycled in the previous step using a high C-rate (2C), and subsequently was cycling using a low C-rate (0.1C). A conclusive explanation might be that lithium remaining in the Si/C electrode during the previous cycling (from 0.5C to 2C) could be extracted at 0.1C. By laser structuring of electrodes, the stability of coulombic efficiency and capacity retention depending on the applied C-rate could be successfully improved in comparison to cells with unstructured electrodes.

In addition, the discharging process, namely, lithiation in anode material in a half-cell, is related to the charging process in a full-cell. Figure 4c,d presents the discharging capacity associated with time and voltage. This result can represent the efficiency of the charging process in real full-cells. At 0.1C, a capacity of 473 mAh/g was achieved by reference cell applying constant current (LS cell: 494 mAh/g), while the cell reached the cutoff voltage of 0.01 V. After the cccv (constant current–constant voltage)-discharging procedure, both cells delivered similar capacities of 498 mAh/h and 510 mAh/g, respectively. By applying 1C, the voltage-capacity behaviour changed dramatically. The voltage of the reference cell dropped within 3.4 min to 0.01 V with a capacity of 30 mAh/g, while the LS cell reached the cutoff voltage after 17.2 min, providing a capacity of 152 mAh/g. Eventually, the LS cell could achieve almost double capacity (404 mAh/g) in comparison to the reference cell (220 mAh/g). It can be determined on the basis of the obtained results that the lithiation process mainly took place during the constant-voltage step by applying 1C. The rapid voltage drop of the reference cell within a few minutes indicated a distinct cell polarization (also called overpotential) at high C-rates. The kinetically limited diffusion of the lithium-ions caused the cell overpotential and the lithium concentration gradient within the porous electrode, which can be demonstrated by applying laser-induced breakdown spectroscopy.

#### 3.2.3. Lithium Distribution and Visualisation of Lithium Diffusion Pathway

**Figure 5:**
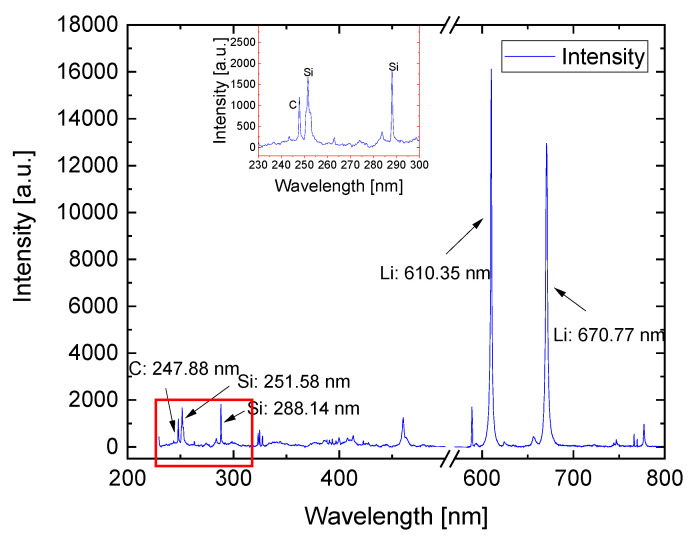
LIBS spectrum of a silicon–graphite electrode with the indication of the most relevant emission lines of lithium, graphite, and silicon.

The LIBS measurement was performed on the cycled Si/C electrode, and a typical spectrum is illustrated in Figure 5 with marked relevant characteristic emission wavelengths of the elements for the Si/C electrodes. The intensity of a characteristic wavelength enables qualitative analysis of the respective element and its distribution in the entire electrode. Due to the self-absorption effect for the most dominant Li^I^ emission wavelength at 670.77 nm, the Li^I^ emission wavelength at 610.35 nm was chosen for the analysis of lithium [26].

**Figure 6:**
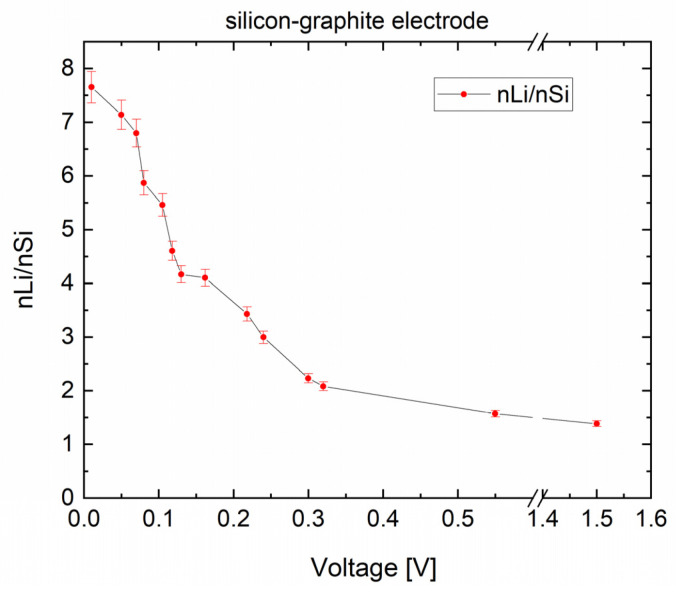
Relative content of lithium to silicon (nLi/nSi) in silicon–graphite electrodes measured by inductively coupled plasma optical emission spectroscopy (ICP-OES) as a function of depth of discharge.

The element mass ratio of Si/C calibration samples was determined by ICP-OES, and the lithium–silicon ratio (nLi/nSi) was calculated as a function of voltage (Figure 6). The nLi/nSi ratio decreased as expected with increasing voltage. The maximum lithium–silicon ratio (nLi/nSi) was 7.65 ± 0.29 for the sample lithiated to 0.01 V. This value was summed up by the lithium amount in silicon, graphite, and SEI layer. In the fully delithiated sample charged to 1.5 V, the nLi/nSi-ratio was 1.38 ± 0.05. Inactive lithium remaining in SEI and irreversible alloyed Si-Li mainly contributed to this measured lithium content. LIBS measurements were subsequently performed on the calibration samples. The measured spectra were analyzed by Software Sec Analysis Tool (SECOPTA analytics GmbH, Teltow, Germany) with regard to the element mass ratio (Figure 6). A correlation between the ratios of intensities of spectra emitted by elements and the quantitative ratios of amounts of substance of elements measured by ICP-OES was established. In addition, a validation process was carried out in order to ensure the accuracy of the established calibration curve.

**Figure 7:**
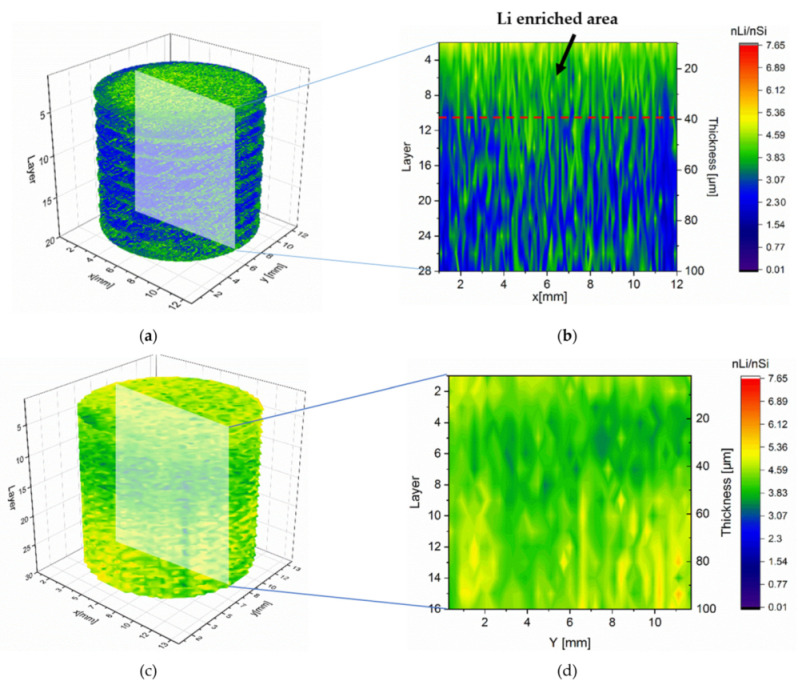
Lithium concentration profile in entire structured and unstructured Si/C electrodes lithiated at 1C: (**a**) 3D illustration of lithium concentration in a reference electrode, (**b**) its lithium distribution in the cross-sectional view, (**c**) 3D illustration of lithium concentration in a structured electrode (pitch distance: 300 µm), and (**d**) its lithium distribution in the cross-sectional view.

Lithium concentration and homogeneity were investigated post-mortem at the reference and LS electrodes. The studying electrodes were discharged at 1C to 0.01 V, and the cell voltage was kept to 0.01 V for 10 min. As a comparison, a reference electrode without laser structuring was discharged by the same procedure but using a lower C-rate of 0.05C. The LIBS measurements were performed on the surface of the unstructured Si/C electrode by applying so-called elemental mapping with a point-to-point distance of 100 μm. The material was ablated layer by layer down to the current collector. With regard to the size of the resulting laser ablation crater profile on the electrode material (diameter > 200 µm), the grid structures with a pitch distance of 300 µm were utilized for LIBS measurements. The LIBS pulses were positioned in the center of the grid structures. The lithium concentration in this work was defined as the ratio of the amount of Li substance to that of Si (nLi/nSi). This value obtained from the cross section of the reference electrode was 6.34 with a standard deviation of 0.63. Figure 7 presents the elemental mapping of lithium distribution and concentration within an unstructured and structured Si/C electrode from the top of the electrode down to the current collector every four layers and the cross-sections at the position y = 6.4 mm within the entire electrode. An inhomogeneity of lithium distribution in the unstructured is shown in Figure 7a,b, where a lithium enrichment on the top (about 40 µm) of the electrode was detected. The average lithium concentration of the selected cross section was 3.38, with a standard deviation of 0.80, which corresponded to a measured specific capacity of 212 mAh/g. Figure 7b exhibits the resulting lithium concentration profile as a consequence of lithium-ion diffusion through the separator and gradually diffusion from the outer electrode surface into the bulk until it reached the current collector. The lithiation process was implemented gradually and locally from the top of the electrode to the bottom. Contrary to this observation, the structured electrode revealed a higher average lithium concentration of 4.30 and a significantly enhanced homogenized lithium distribution in the entire electrode with a standard deviation of 0.42 (Figure 7c,d). This result corresponded to a higher measured specific capacity of 272 mAh/g. Laser structuring shortened the diffusion path of the lithium-ions and reduced the overall cell impedance, which led to a significant increase in diffusion kinetics at high C-rates.

**Figure 8:**
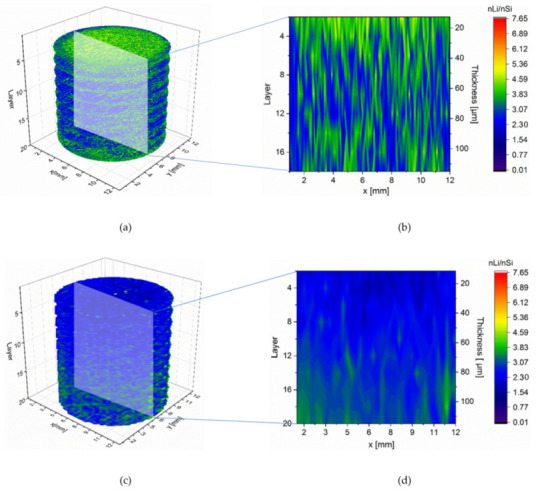
Lithium concentration profile in entire structured and unstructured Si/C electrodes delithiated by 1C: (**a**) 3D illustration of lithium concentration in a reference electrode, (**b**) its lithium distribution in the cross-sectional view, (**c**) 3D illustration of lithium concentration in a structured electrode (structure distance: 300 µm), and (**d**) its lithium distribution in the cross-sectional view.

A dramatic increased coulombic efficiency was obtained in the first cycle when the operation current was changed from high C-rate (2C) to low C-rate (0.1C). The reason could be experimentally explained by means of LIBS measurements on the delithiated electrodes. The lithium concentration of the structured and unstructured electrodes after delithiation at 1C is shown in Figure 8. The studying electrodes were lithiated with a low C-rate of 0.2C to ensure a complete lithiation and subsequently were delithiated at 1C to 1.5 V, keeping for 10 min. In the unstructured electrode, an average lithium concentration of 3.27 ± 0.75 was detected. Due to the laser-generated new diffusion pathways along the channel sidewalls, the lithium-ions could efficiently be extracted from the electrode material and revealed a lower average concentration of 2.70 ± 0.45. The residual lithium in the electrode, which could not be completely extracted from the host material, led to the capacity fading at high C-rates. The process is particularly reversible, and the residual lithium in the electrode can be extracted by applying low C-rates, which can explain the extremely high coulombic efficiency in Figure 4b after switching to a low C-rate. 

**Figure 9:**
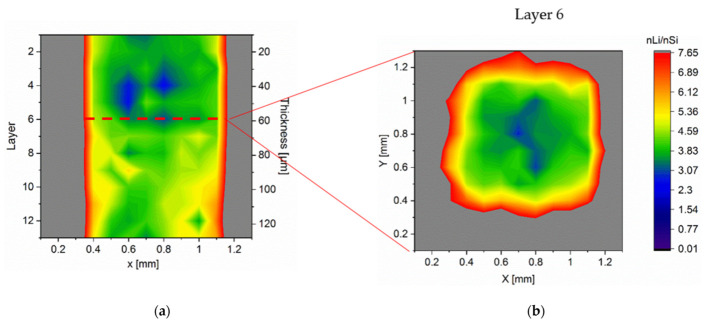
Lithium distribution of a Si/C micropillar lithiated at 1 C: (**a**) cross-sectional concentration profile of the micropillar at position y = 0.7; (**b**) horizontal layer 6 of the micropillar cross-sectional mapping.

The achieved results indicate that laser-generated 3D electrodes can accelerate the lithium-ion transport during the lithiation and delithiation process and thereby realize the fast charging with reduced capacity loss. The lithium diffusion pathways established at a high C-rate (1C) were further investigated in detail using electrodes with micropillar structures. The cells with model electrodes (see Figure 1) were prepared in the identical way (Table 3). The micropillars were ablated layer-by-layer by LIBS laser pulses with 100 µm offset. The lithium distribution in a selected micropillar lithiated with 1C is presented in Figure 9. From the cross-sectional view (Figure 9a), it is obvious that a lithium enrichment with values nLi/nSi > 6 was located along the contour of the micropillar. Layer 6 of the horizontal cross-section (Figure 9b) revealed a minimum lithium concentration in the center of the micropillar.

Oumellal et al. [10] describe how lithium-ions diffuse from the separator via the pores inside the electrode filled with liquid electrolyte into the particles. The LIBS measurements indicate that at high C-rates the lithium-ions prefer to diffuse through the free electrolyte via laser-generated sidewalls of micropillars. This is mainly due to two reasons. Firstly, the lithium-ion mobility in free electrolyte is faster than in the composite electrode, which is affected by porosity and tortuosity. Secondly, the 3D topology of composite electrodes contributes to the formation of lithium-ion diffusion pathways. During intercalation, lithium-ion diffusion takes place between the basal planes of graphite. Therefore, the graphite electrodes have different tortuosity values parallel and perpendicular to the basal plane [34]. After coating, drying, and calendaring, the disc-shaped graphite particles are stacked on top of each other with a preferred orientation parallel to the current collector [35,36].

However, in contrast to the observation on graphite micropillars in our previous work [37], lower lithium concentration (nLi/nSi ≈ 3.4) was detected on top of the electrode (layer 1–layer 6). This was attributed to the compressive pressure in the electrodes caused by the volume expansion of silicon. The compressive stress was forced on the separator and caused closure of pores inside the separator. Meanwhile, due to the volume expansion of silicon, the porosity inside the electrode was also reduced. As mentioned in Section 4.1, the lithiation process can be restricted by mechanical compressive stress. The electrode materials along the sidewalls were free from mechanical stress. Hence, lithiation, especially lithiation in silicon, was effectively promoted there.

#### 3.2.4. Post-Mortem Analysis

**Figure 10:**
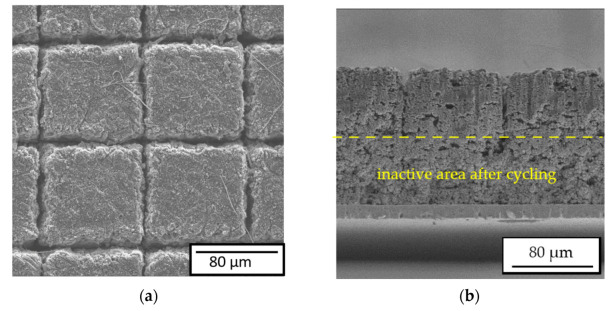
SEM images of cycled Si/C electrodes: (**a**) top view and (**b**) cross-sectional view.

Laser-generated channels can significantly reduce the overall mechanical stress inside Si/C-electrodes [25]. The cells in Figure 4 were dissembled in the delithiated state for a post-mortem SEM analysis. Figure 10 illustrates the SEM images of the cycled structured Si/C electrode, which can be geometrically divided into two parts. Due to the volume expansion, the laser-generated V-shaped channels (Figure 2b) were deformed. Only on the top half of the electrode, a small channel structure could still be detected. In the lower half of the electrode, close to the current collector, the channels were filled due to irreversible volume expansion, namely, chemical degradation [10]. Concerning the established channel geometry in Figure 2, one can state that the minimum channel width should be larger than 15 µm regarding the volume expansion. Increased capacity retention and cell lifetime of the same electrodes can be achieved by using wider channels (not shown here). The main degradation mechanism of the Si/C electrode can be described by a combination of mechanical and chemical degradation.

**Figure 11:**
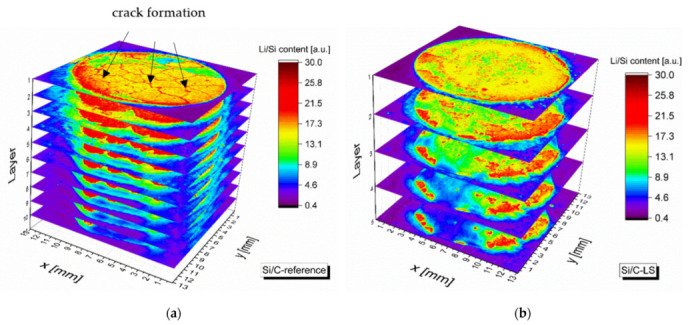
Post-mortem qualitative analysis of the electrochemically cycled electrodes by LIBS: (**a**) reference electrode without laser patterning and (**b**) laser patterned electrode.

LIBS can be used as post-mortem analysis to investigate the impact of cell degradation and to draw conclusions about the degradation mechanisms. The qualitative analysis of 3D lithium concentration of the reference electrodes without laser structuring and laser-structured electrodes was performed as described in [25] and is shown in Figure 11. A lack of film adhesion combined with the volume expansion resulted in film delamination and crack formation on the reference electrode, which can be impressively shown on the first layer in Figure 11a. A higher lithium concentration was detected in the entire reference electrode than in the LS electrode. Therefore, the reference electrode had a higher material density due to the higher Li content than the LS electrode. As a result, the reference electrode revealed a lower laser ablation rate of ≈ 5.5 µm per layer, which is defined by the ablation depth per layer and corresponds to the LIBS information depth. In contrast, the laser ablation rate of the cycled LS electrode was higher (≈11 µm per layer) due to lower lithium concentration.

The 3D mapping extracted from the qualitative analysis of the lithium concentration (Li/Si) indicates that the lithium transport was restricted due to long diffusion pathways from the current conductor to the counter electrode. The residual amount of inactive lithium inside the electrode increased cycle by cycle and led to an irreversible capacity fading and volume expansion. Finally, cell failure occurred.

Since the lithium could also reach the counter electrode via the sidewall of the freestanding structures, the lithium content in the LS electrode was found to be lower than that in the reference electrode. The reason for the cell degradation with Si-based electrodes is the lithium irreversibly stored in the electrode. Lithium-ions could not be completely extracted from the electrode materials during cycling. This correlates with the cell voltage of the cyclized cells in a delithiated state. After the long-term tests, the open-circuit voltage (OCV) of the half-cells in the delithiated state was mostly below 200 mV. The OCV of fresh cells is usually around 900 mV.

## 4. Discussion

### 4.1. Impact of Mechanical Stress on the Phase Change

The phase changes in silicon and Si/C electrodes were investigated by cyclic voltammetry. The current peak at 0.45 V in the anodic reaction (delithiation) can be regarded as a material-specific indicator for the formation of cr-Li_15_Si_4_. A wide range of low cutoff voltage (0.03 V–0.08 V) was reported with regard to the formation of this crystalline phase. Hatchard and Dahn [38] reported that amorphous Si films crystallize below 0.03 V, and the c-Li_15_Si_4_ phase does not form when the film thickness is larger than 2.5 µm. In this paper, the crystalline phase could be only detected in LS electrodes when the lower cutoff voltage was lower than 0.05 V. The LIBS studies of lithium distribution in structured and unstructured graphite and Si/C electrodes showed that lithium preferentially intercalated into the electrodes via the laser-generated sidewalls. The obtained results from CV and LIBS indicated that compressive stress affected both the electrochemical potential and the kinetics of the electrochemical reaction. The CV experiments revealed that cutoff voltage is not the only condition for crystallization. Compressive stresses within the electrode can reduce the electrochemical potential and restrict the formation of the crystalline phase, namely, full lithiation. 

### 4.2. Degradation Mechanisms of Silicon–Graphite Electrodes

Oumellal et al. [10] investigated the silicon electrode failure mechanism using nuclear magnetic resonance (NMR) spectroscopy. This showed that most of the lithium lost during battery charging is not trapped in the Li_x_Si alloys but adheres to the surface of the Si particles. Therefore, a thick SEI layer formed on the surface of the failing electrodes, which was probably produced as a degradation product of the liquid electrolyte during cycling. The groups of Peled and Aurbach [12,39] investigated the SEI-related side reactions that occur at cyclized negative electrodes of lithium batteries. These works show that lithium-ions are irreversibly trapped by oligocarbonate molecules that deposit in the electrode pores. One of the most important side reactions in a medium with high ethylene carbonate (EC) concentration could be related to a nucleophilic ring-opening of the EC molecules by the reduced Si surface [10].

**Figure 12:**
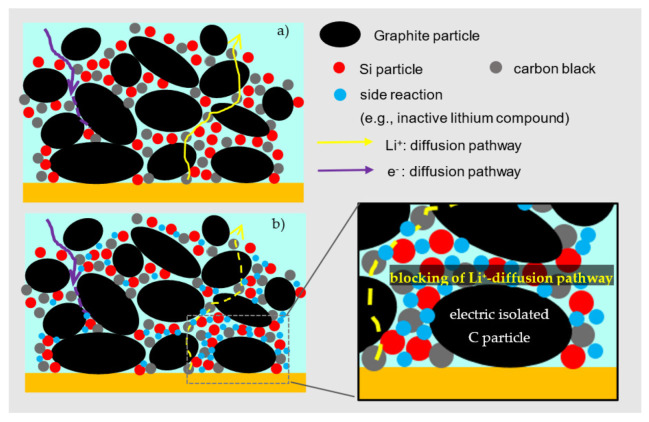
Schematic representation of the silicon–graphite electrode and electron diffusion pathways within the composite electrodes: (**a**) in a fresh state and (**b**) in a cycled state.

Nevertheless, not only chemical degradation causes the capacity fading. Due to the trapped lithium on the silicon surface, the porosity of electrodes was also reduced. As shown schematically in Figure 12, the lithium-ions in a fresh Si/C electrode can diffuse through the pores to the silicon particles and conversely diffuse to the separator in the same way during delithiation. The degradation products are deposited in the pores and on the surface of the silicon particles during cycling. On the one hand, side products reduce the porosity and meanwhile extend the diffusion paths of the lithium-ions cycle by cycle. On the other hand, the reduced porosity leads to an increase in mechanical stress within the electrode. Crack formation in Si particles could be caused by the pressure of the surrounding materials, and new SEI layers are gradually rebuilt.

The standard graphite electrodes were able to achieve a cycle lifetime of up to 1000 cycles in the full cell format and showed capacity retention of 80% after testing. The LS silicon electrodes with 40 wt% silicon exhibited a longer cycle life than the Si/C electrode and could still deliver half of the initial capacity after 400 cycles (shown in the Appendix A). The significantly reduced capacity retention and lifetime of Si/C electrode can be attributed to the blocking of the lithium-ion access to graphite particles (Figure 12b). Since the silicon nanoparticles and conductive carbon black were homogeneously placed between the macroscale graphite particles with the help of the binder, the graphite particles were surrounded by silicon particles. Due to the chemical degradation, the porosity of the Si/C electrode was reduced by irreversible volume expansion and side products. The lithium trapped on the silicon surface was no longer available for the electrochemical reaction, and meanwhile, the inactive materials reduced the active surface area of the surrounding graphite particles. Finally, the diffusion access on the graphite surfaces to the liquid electrolyte was blocked (Figure 12b). As a result, graphite particles were ionically and electrically isolated.

By means of LIBS measurements, less lithium was detected in the structured electrode. The chemical degradation was correlated by mechanical stress and decelerating diffusion transport due to reduced porosity and increased tortuosity. Laser structuring on Si/C electrodes can significantly reduce mechanical stress and provide new diffusion accesses to the bulk electrode material. Therefore, the lower Li content of the LS cell by a factor of about 1.5 showed that laser structuring can significantly contribute to reducing cell degradation. In addition, on the basis of the results obtained from post-mortem analysis in Figure 10, we found that the geometry of channels affected the degradation process as well. Lack of free spaces on the bottom of the structured electrode (Figure 2b) might elicit a more severe cell degradation in comparison to it occurring on the top of the electrode. The pores there might be completely blocked and result in advanced cell failure. Therefore, cells with entire channel width larger than 15 µm revealed improved capacity retention and cell lifespan.

## 5. Conclusions

In this work, the high-rate capability and cycle stability of cells with structured and unstructured Si/C electrodes were presented. The impact of degradation processes in Si/C electrodes was correlated in relation to the lithium concentration profiles in the entire electrodes. The lithium concentration and distribution of the structured electrodes revealed that at elevated C-rates, new lithium-ion diffusion pathways into the composite electrodes were established along the laser-generated sidewalls. The observed effect was due to low compressive stress in the active material along the sidewall and a high lithium-ion mobility in the free electrolyte in comparison to the electrolyte embedded in the porous composite electrode. The cell degradation of Si/C electrodes was attributed to the trapped lithium in the Si/C electrode. By means of post-mortem analysis of the morphology and the lithium concentration obtained from LIBS measurements, we can conclude that the mechanical stress can also accelerate chemical degradation and lead to early cell failure. 3D architectures on the Si/C electrodes enable a high rate capability, accelerate the lithium-ion transport, and can effectively reduce the mechanical stress as well as cell degradation. All those benefits can push silicon-based electrodes beyond the state of the art to become a high-energy anode material for next-generation batteries.

## Figures and Tables

**Table 1:** Chemical composition and theoretical capacity of the prepared silicon and Si/C electrodes.

Type	Composition (wt%)					Areal Capacity * (mAh/cm²)	Thickness (µm)	Analytics
	Si	Graphite	Carbon	CMC	SBR			
1	40	0	30	15	15	2.42 ± 0.13	25 ± 2	CV
2	20	60	10	5	5	3.09 ± 0.20	55 ± 2	LIBS
3	10	70	10	5	5	4.03 ± 0.05	70 ± 2	Calibration
4	10	70	10	5	5	6.44 ± 0.10	100 ± 2	LIBS and GM

* related to reference electrode.

**Table 2:** The electrochemical titration voltages of cells prepared for LIBS calibration.

Electrochemical Titration Voltages (V)						
0.01	0.05	0.08	0.105	0.118	0.13	0.162
0.2	0.218	0.24	0.3	0.32	0.55	1.5

**Table 3:** Electrochemical testing procedures for the investigation of lithium concentration profiles.

Samples	Cell State for LIBS	Step 1 (formation)	Step 2	Step 3
(a) Unstructured Si/C electrode (b) LS Si/C electrode (c) Si/C model electrode	lithiated state at 0.01 V (0.05C)	discharging/charging: 0.02C; cycles: 1	-	discharging: 0.05C cv at 0.01 V for 10 min
	delithiated state at 1.5 V (0.05C)		discharging: 0.05C	charging: 0.05C, cv at 1.5 V for 10 min
	lithiated state at 0.01 V (1C)	discharging/charging: 0.05C; cycles: 2	-	discharging: 1C, cv at 0.01 V for 10 min
	delithiated state at 1.5 V (1C)		discharging: 0.2C	charging: 1C, cv at 1.5 V for 10 min

**Figure A1:**
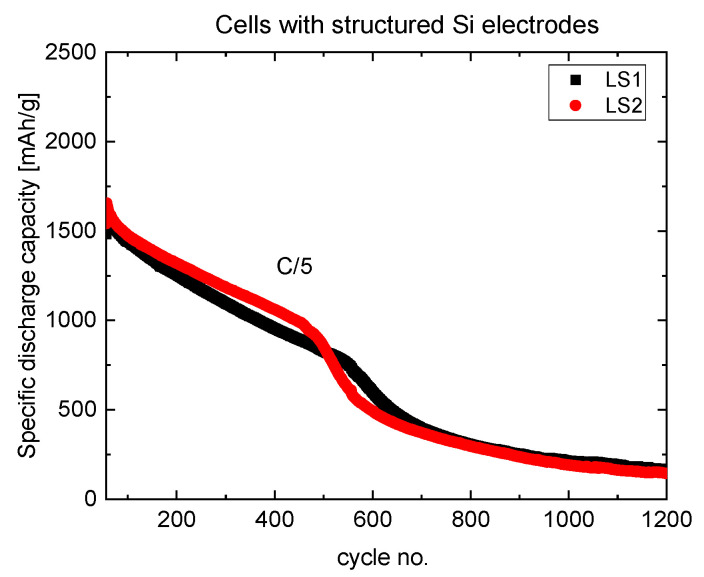
Galvanostatic result of cells with structured (LS) silicon electrodes (type 1 in Table 1).

## Data Availability

Data sharing is not applicable to this article.
